# Serological investigation of *Coxiella burnetii* infection (Query fever) in livestock in Makkah Province, Saudi Arabia

**DOI:** 10.14202/vetworld.2024.842-847

**Published:** 2024-04-19

**Authors:** Naser A. Alkenani, Hassan M. Baroom, Adi A. Almohimeed, Salaheldin O. Hassan, Mohammed S. Mohammed, Layla A. Alshehri, S. A. Alshehri, Sulaiman M. Abu Sulayman, Saleh M. Al-Maaqar, Majed A. Alshaeri

**Affiliations:** 1 Department of Biological Sciences, Faculty of Science, King Abdulaziz University, P.O. Box: 80203, Jeddah, 21589, Saudi Arabia; 2 Environmental Protection and Sustainability Research Group, Faculty of Sciences, King Abdulaziz University, Jeddah, Saudi Arabia; 3 Department of Microbiology, Faculty of Science, Umm Alqura University, Makkah, Saudi Arabia; 4 Department of Microbiology, Jeddah Islamic Port Veterinary Diagnostic Laboratory, Ministry of Agriculture, Jeddah, Saudi Arabia; 5 Department of Parasitology, Jeddah Islamic Port Veterinary Diagnostic Laboratory, Ministry of Agriculture, Jeddah, Saudi Arabia; 6 Faculty of Education, Department of Biology, Albaydha University, Al-Baydha, Yemen

**Keywords:** *Coxiella burnetii*, enzyme-linked immunosorbent assay, livestock, Saudi Arabia, serology

## Abstract

**Background and Aim:**

Query fever (Q fever) is an endemic zoonotic disease and ruminants are considered to be the primary source of infection in humans. It is caused by *Coxiella burnetii* which is an obligate intracellular bacterial pathogen with a worldwide distribution. This study estimated the prevalence of Q fever in livestock with a history of abortion in Makkah Province, Saudi Arabia.

**Material and Methods:**

Sera from 341 camels, 326 sheep, and 121 goats of either sex from various locations (Makkah, Jeddah, AL-Taif, AL-Qunfudah, AL-Laith, and AL-Kamil) were examined using a Q fever indirect enzyme-linked immunosorbent assay.

**Results:**

Among the 788 serum samples, 356 animals had anti-*Coxiella burnetii* immunoglobulin G antibodies with an overall seroprevalence of 45.4%. Significant differences were observed in seroprevalence between species and locations. Camels had the highest percentage of Q fever-positive sera, with a prevalence of 50.4%, followed by goats (44.6%) and sheep (36.8%), with a high significant difference between animals (p = 0.000). The prevalence was significantly higher in Makkah (65.4%) than in Jeddah (28.8%).

**Conclusion:**

*C. burnetii* infection is prevalent in agricultural animals, especially camels maintained at livestock farms in Makkah province. Therefore, these animals considered as the main source of Q fever infections in Saudi Arabia, which is also a reason for the abortion in these animals. Therefore, there is an urgent need for further studies on Q fever infection with interventional approaches for prevention and control.

## Introduction

Query fever (Q fever) is a zoonotic illness [1]. It is caused by the obligatory intracellular bacterial infection *Coxiella burnetii*, which has a widespread distribution and may survive in the environment for a long period of time or may travel great distances by wind [2]. Ruminants are believed to be the main source of infection for humans [3]. Q fever is usually transmitted to humans through the inhalation of contaminated aerosols and livestock products, such as raw milk, or through contact with the fetus [4–6], although sexual transmission between persons is uncommon [7].

Ticks are now regarded as a source of *C. burnetii* infection in the environment, which is thought to be limited to passive transmission in the surroundings [8]. In domestic ruminants, the disease syndrome appears as miscarriage, sterility, mastitis, and stillbirths [9], whereas in humans, it appears either acute as a relatively minor self-limiting febrile sickness or a chronic more severe disease characterized by hepatitis, pneumonia, and chronic fatigue [10].

Q fever was first identified as holo-endemic among Saudi Arabian citizens in the 1960s [11, 12], when Q fever was first mentioned in Saudi Arabia. Among 51 Saudi Arabians, 18 were positive for antibodies to Q fever by immunofluorescence testing [13]. There is a lack of knowledge regarding Q. fever in livestock in Saudi Arabia, which has been observed in sheep, goats, cattle, and camels in various sections of the country [14], especially in pastoral areas where animals are the main source of income and in deserts for wild ungulates [15]. In 1995, 10.6 seroconversions occurred every 1000 person-months among American soldiers returning from Saudi Arabia after serving in Iraq, most likely due to contact with animals there [16]. Several studies in Saudi Arabia have shown that a large percentage of abortion animals, such as camels, cattle, sheep, gazelles, and goats, have Q fever infection [17–20].

Because farm livestock infected with *C. burnetii* sometimes experiences reproductive disorders, besides being the main source of human infection, especially in Saudi Arabia, serological investigations of these diseases may reveal the diagnosis, early detection and control of disease, and public health protection in the future for these diseases (Q fever) in livestock, causing an increase in the elimination of Q. fever in the future.

This study aimed to use indirect enzyme-linked immunosorbent assay (ELISA) to estimate the prevalence of Q fever (*C. burnetii*) in livestock from different localities in Makkah Province, Saudi Arabia.

## Materials and Methods

### Ethical approval

This study was approved by the research commenced from the King Abdulaziz University, Faculty of Science Biosafety, Animal Care, and Use Committee (Approval no. RHKB7796- 8002472220). Permission was requested and granted by the Animal Resources Sector of the Ministry of Environment, Water and Agriculture (MEWA), Jeddah, Saudi Arabia to undertake the research. The investigation was carried out in compliance with the Animal Welfare Act (system) for the States of Cooperation Council for Arab States of the Gulf (Chapter 2; Article 2).

### Study period and location

**Figure-1:**
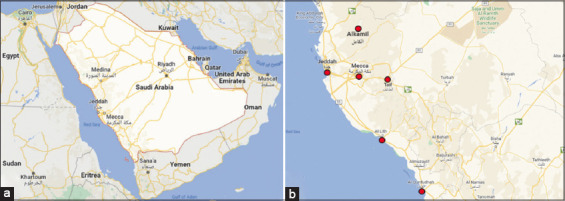
(a and b) Sample collection area in Makkah region, Saudi Arabia.

The study was conducted from October 2021 to October 2022 at six ecologically Makkah region (Makkah, Jeddah, Taif, Qunfudah, Laith, and Kamil) areas in the western region of Saudi Arabia (Figure-1), which is considered the largest area and most populous in Saudi Arabia [21].

### Blood sample collection

Blood (788) samples were collected randomly from apparently healthy animals (341 camels, 326 sheep, and 121 goats) of both sexes and different ages from a national educator’s farm in some areas of the Makkah Region in which cases of abortions had previously occurred. The sex, age, or dynasty of the collected animals was not seen because the goal was to investigate (i.e., the spread of the disease) (Table-1). Approximately 3–5 mL of blood was collected from the jugular vein into vials using plain vacutainers and needles. The coagulated blood was then centrifuged at 1500× *g* for 10 min in the laboratory. Hemolysis is replaced and eliminated. A serological examination was performed for anti-*C*. *burnetii* antibodies using enzyme-linked indirect immunosorbent assay (ELISA) from farm animals with a history of abortion.

**Table-1:** Blood samples collected from animals in different localities in Makkah Province, Saudi Arabia from 2021 to 2022.

Locality	Camel	Sheep	Goats	Total
AL-Taief	103	101	58	262
AL-Laith	89	78	36	203
Jeddah	67	111	13	191
Makkah	17	2	7	26
AL-Qunfudah	41	22	7	70
AL-Kamil	24	12	0	36
Total	341	326	121	788

0=Not taken

### Sample size determination

In determining the sample size, the following formula [22, 23] was used to calculate the minimum number of camel, sheep, and goats for the study: n = Z^2^ P_exp_(1 – P_exp_)/d^2^, where n is the required sample size, P_exp_ is the expected prevalence, d is the desired absolute precision, Z is the normal deviation (1.96) at 95% confidence level, P is the estimated prevalence (p = 30% proportion level prevalence of 30% which was considered as in a previous study by Jarelnabi *et al*. [18] in Saudi Arabia, and d is the precision of the estimate. A sample size of 323 was required when P was set at 0.03 and d at 0.05.

### ELISA

CHEKIT-Q-Fever (IDEXX Laboratories, Bommeli Diagnostics AG, Lieberfeld-Bern, Switzerland) was used as the commercial ELISA Q fever antibody test kit. Each kit consisted of a microtiter plate with 96 flat-bottomed wells pre-coated with deactivated *C. burnetii* antigen, monoclonal anti-ruminant immunoglobulin G (IgG) antibodies attached with horseradish peroxidase, reference sera from both negative and positive controls, a 10-fold concentration of phosphate-buffered normal saline, tetramethylbenzidine chromogen substrate N12, and stop solution (0.05 mL 2M H_2_SO_4_). This was performed using a monoclonal anti-ruminant IgG combination. Additional test requirements included a 96-well microplate reader, microplate washer, shaker, incubator (37°C), 8- and 12-channel precision pipettes with plastic disposable tips, and distilled water.

The ELISA was performed according to the manufacturer’s instructions. The examination sera, as well as the reference negative and positive control sera, were diluted to a concentration of 1:400 with phosphate-buffered saline, added in duplicate amounts of 100 μL to the wells of the microtiter plates, and gently shaken. The plates were washed three times in a washing solution (300 μL/well each time) at room temperature to remove any loose material from the wells. Plates were covered with plastic lids and incubated in a humid environment at 37°C for 60 min. After the last wash, the plates were lightly tapped to remove any remaining washing solution. Following the addition of 100 μL of freshly produced conjugate into each well, the plates were covered with lids, incubated at 37°C for 60 min, and washed three times in the same washing solution. After adding 100 μL of freshly prepared chromogen substrate solution to each well, the plates were gently shaken and left at room temperature (20°C–22°C) for 15 min. A microplate reader (MTX Labsystem Inc., 8456 Tyco Road, Vienna, VA 22182, U.S.A.) was used to measure the absorbance of each well at 450 nm, following which the color reaction was stopped by adding 300 μL/well of the stop solution.

The manufacturer reported the sensitivity and specificity of the CHEKIT-Q-Fever IDEXX ELISA kit to be approximately 100% [24]. This test is approved for use in sheep, goats, and cattle. IDEXX ELISA is commonly used in serum samples of camels; however, it has not been validated in camelids [25, 26]. The absorbance data were then used to calculate the results. The results are expressed as a percentage of the optical density (OD) reading of the test specimen (% OD), which is calculated as %OD = 100 × (S–N)/(P–N), where S, N, and P are the OD readings of the test specimen. If the OD was <40, it was considered doubtful; if it was between 40 and 50, it was considered positive; if it was >80, it was considered very positive.

### Statistical analysis

The data collected were analyzed using the Statistical Package for the Social Sciences version 26 (IBM Corp., NY, USA). The data were subjected to analysis of variance using the Chi-square test (χ^2^) procedure according to Gomez and Gomez [27] to compare differences between the means of different regions and species. Differences between the analyzed parameters were investigated at p < 0.05 probability value levels.

## Results and Discussion

Serum samples from agricultural animals (camels, sheep, and goats; Table-1) were examined to investigate the prevalence of *C. burnetii*-specific IgG antibodies in farms of animals with a history of abortion. Among the 788 serum samples, 356 animals had anti-*C. burnetii* IgG antibodies, resulting in a serological prevalence of Q fever of 45.4% (Table-2).

**Table-2:** Prevalence of *C. burnetii* antibodies in animals collected from different localities in the Makkah region, Saudi Arabia, from 2021 to 2022.

Location	Camels		Sheep		Goats		Overall prevalence		p-value
			
	No. tested	+ve (%)	No. tested	+ve (%)	No. tested	+ve (%)	No. tested	+ve (%)	
AL-Taief	103	65 (63.1)	101	55 (54.5)	58	30 (57.4)	292	262 (57.3)	<0.000
AL-Laith	89	46 (51.7)	78	22 (28.2)	36	14 (38.9)	203	82 (40.4)	<0.000
Jeddah	67	19 (28.4)	111	34 (30.6)	13	2 (15.4)	191	55 (28.8)	<0.000
Makkah	17	11 (64.7)	2	0 (0.00)	7	6 (85.7)	26	17 (65.4)	0.225
AL-Qunfudah	41	22 (53.7)	22	3 (13.6)	7	2 (28.6)	70	27 (38.6)	<0.000
AL-Kamil	24	9 (37.5)	12	6 (50.0)	0	0 (0.0)	36	15 (41.7)	0.439
Overall	341	172 (50.4)	326	120 (36.8)	121	54 (44.6)	818	346 (45.4)	
p-value	<0.000		<0.000		<0.000				

0=Not taken

The overall prevalence reached 45%, which was slightly higher than that reported by Beggary [28] and Abdel Rahman *et al*. [29], who reported prevalence rates of 33.4% and 30.71%, respectively, in the Hail and East regions, Saudi Arabia. Knobel [30] observed a similar finding in goats in Kenya. However, the prevalence varied between species and for the same species in different locations. They pointed out that it could be partly related to regional variations in management and weather conditions.

Camels had the greatest percentage of Q fever-positive sera, with a prevalence of 50.4% out of 341 samples, of which 172 were positive (Table-2). In other studies [29, 31, 32], even higher prevalence (approximately 51%–62%) was observed in Saudi camels. Similarly, camels have a high prevalence rate of 66% in Egypt [33], 29% in Iran [25], 71.2% in Algeria [34], 80% in Chad [35], 44% in Tunisia [36], 31.3% in Pakistan [26, 37] and Eastern Ethiopia [38], as well as in 100% of nomadic camels. This high prevalence may be attributed to poor hygiene and management conditions in which animals live, where crowding and lack of disinfection lead to the transmission of infection, as well as direct exposure to contaminated dust and heat, as well as an appropriate environment for the breeding of ticks that transmit the disease through saliva or feces. Differences between nations may result from many ecological, social, cultural, behavioral, and economic circumstances, as well as different levels of animal diseases, all of which have an impact on how exposed people are in every part of the world [35]. In addition, variations in the percentage of the population engaged in farming activities can be used to explain these variances [36].

According to our findings, camels may be a source of *C. burnetii* in Saudi Arabia and may play a role in the spread of Q fever to people in this region of the world [18]. In addition, Q fever has been frequently reported in camels from other parts of Asia and North Africa [25, 37, 39]. In addition, the genetic susceptibility of camels to *C. burnetii* infection [30, 34] or predilections of tick vectors to camels may have contributed to this phenomenon.

**Figure-2:**
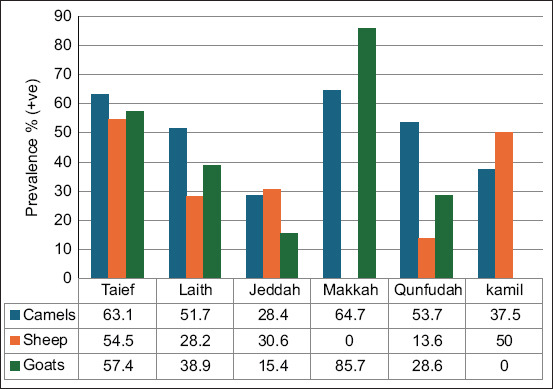
Seroprevalence of *Coxiella burnetii* in different animals according to the region in Saudi Arabia.

Goats had the highest prevalence (44.6%), followed by sheep (36.8%), with significant differences between animals (p = 0.000) (Table-2 and Figure-2). Out of 121 samples, 54 were positive in goats, and in sheep out of 326 samples, 120 were positive. This study is consistent with previous studies by Larson [40], who reported that the highest prevalence rate was in camels, which is consistent with our study, where the percentage was 19.9% of the total 312 samples, of which 62 were positive, followed by goats by 18.2% of the 280 samples, of which 51 were positive, followed by sheep with a percentage 13% of 100 samples, of which 13 were positive.

On the other hand, animals’ geographic locations were also compared, and it was shown that there were significant regional variations in the serological prevalence of Q fever, with Makkah having the highest prevalence of 65.4% and Jeddah having the lowest prevalence of 28.8%, with significant differences between animals (p = 0.000) except in Makkah (p = 0.225) and Kamil (p = 0.439) being non-significant differences between all animals (Table-2 and Figure-2). Furthermore, the seroprevalence of *C. burnetii* in different animals was generally in the following order: Camels > Goats > Sheep. The seroprevalence of *C. burnetii* was characterized at the different sites (Makkah > AL-Taief > AL-Kamil > AL-Laith > Jeddah) (Figure-2).

Makkah recorded the highest prevalence (65%), which is due to hot weather, and Jeddah recorded the lowest (28.8%), which may be due to its humid atmosphere (Table-2 and Figure-2). In Taif, camels and sheep had the highest percentage of positive samples, while sheep had the highest percentage of positive samples in Makkah. Similarly, camels have a high prevalence rate; 40.7% in Egypt [33], 73% in Chad [35], 46% in Kenya [40], and 28.7% in Iran [41]. The high prevalence of *C. burnetii* in camels in the Makkah region is somewhat concerning and may be due to several factors, including the large increase in the number of camels and small ruminants, where they are in large numbers, in a narrow, unprepared place, and poor hygienic breeding conditions, in addition to the polluted sand and unfit for drinking water, as well as the large presence of ticks on different parts of the body of camels. Furthermore, *C. burnetii* was easily transmitted by the wind to large areas, resulting in the inhalation of contaminated aerosols from the amniotic fluid, placenta, or contaminated wool, direct consumption of the placenta or milk of infected animals, or tick bites [42–44]. Moreover, it is possible that consuming camel milk without pasteurization plays a significant role in the spread of Q fever to humans. This is followed by goats, which account for a large proportion of camels because they graze around dwellings, which increases the risk of infection. These factors contribute to and increase the risk of *Coxiella* infection in the region.

The results published may help local governments decide which control measures, such as the introduction of animal vaccination, the protection of workers at higher risk of infection, or tick management, can be achieved through acaricide use, as well as hygienic measures that can reduce Q fever contamination and spread and minimize movement of *C. burnetii* from small to large ruminants. Treatment of Q fever in animals is based on farm management strategies and prevention measures rather than chemotherapeutic treatment [13, 17].

## Conclusion

The findings of this study show a high seroprevalence of Q fever (45%) among livestock animals, especially camels, in the Makkah region of Saudi Arabia. This relatively high prevalence suggests that Saudi Arabia is an important endemic focus of Q. fever, which is also a reason for the abortion of these animals. However, *C. burnetii* infections may also occur in other animals and people in the study region. Therefore, it is recommended that further studies on diagnosing Q fever and management procedures should be conducted in livestock husbandry regions in Saudi Arabia.

## Authors’ Contributions

HMB, MSM aud NAA: Conceptualization, methodology, writing original draft, writing review and editing. HMB, SOH and SAA: Data collection and curation. HMB, AAA, SMA and LAA: Conceptualisation, methodology, and carried out the ELISA assays. SMAS. Data collection and curation, MAA and SMA: Carried out data analysis, writing-original draft, All authors have read, reviewed, and approved the final manuscript.
